# Lateral Ankle Ligaments: An Insight Into Their Functional Anatomy, Variations, and Surgical Importance

**DOI:** 10.7759/cureus.53826

**Published:** 2024-02-08

**Authors:** Akhalaq Ahmed, Pravash Mishra, Bishnu Patra, Praveen Kumar Ravi

**Affiliations:** 1 Anatomy, Jawaharlal Nehru Medical College, Aligarh, IND; 2 Anatomy, All India Institute of Medical Sciences, Bhubaneswar, Bhubaneswar, IND; 3 Orthopedics, All India Institute of Medical Sciences, Bhubaneswar, Bhubaneswar, IND

**Keywords:** anterior talofibular ligament, cadaver, ankle joint, lateral ligament complex, ankle sprains

## Abstract

Background: Ankle sprains are prevalent injuries leading to functional impairment. The lateral ankle ligament complex (LLC), comprising the anterior talofibular ligament (ATFL), posterior talofibular ligament (PTFL), and calcaneofibular ligament (CFL), is weak and prone to injury. The morphometric data of these ligaments are essential for orthopedic practices, including techniques like direct repair or ATFL reconstruction with autograft/allograft, which are limited in the literature. The present study aims to document the anatomy and morphometry of the LLC.

Methods: Fifteen adult Indian-origin embalmed cadavers were selected for the study. Ankles with antemortem or postmortem injuries or previous surgical interventions were excluded from the study. After precise dissection of the ankle's anterior and lateral aspects as per Cunningham's dissection manual, ligaments were exposed. Length and width were measured using a digital vernier caliper. Morphological attributes such as shape, orientation, and inter-fiber angles were documented.

Results: The most common shape in ATFL was a single band (53.33%). Inner ATFL fibers merged with the ankle joint capsule in 73.33%. ATFL mean length and width were 14 ± 2.4 mm and 7.6 ± 2.0 mm. The angle between the fibula's long axis and ATFL fibers was 107 ± 22°, and the angle between tibiotalar joint lines and parallel ATFL fibers was 30 ± 9.5°. A single band of CFL was predominant (73.33%). The mean length and width of CFL were 18.4 ± 3.9 mm and 5.2 ± 1.3 mm; the angle between the anterior fibula border's long axes and parallel CFL line was 131°. PTFL length was 20.9 ± 3.3 mm and width was 6.2 ± 1.4 mm. The mean length and width of the anterior inferior talofibular ligament (AiTFL) were 11.7 ± 2.6 mm and 9.5 ± 1.6 mm, and of the posterior inferior talofibular ligament (PiTFL) were 12.8 ± 2.1 mm and 10.4 ± 2 mm.

Conclusion: Comprehensive knowledge of these ligaments' anatomy and relationships is vital for clinical examination and ultrasonography. Understanding LLC details aids radiologists and orthopedic surgeons in graft selection, sizing, and precise anatomical structure placement during surgical reconstruction.

## Introduction

Ankle sprains represent one of the most common types of ankle injuries encountered by athletes, resulting in functional impairment. Ankle ligaments can be broadly classified into three groups: lateral ankle ligaments, medial ankle ligaments, and tibiofibular syndesmosis. Among these, the lateral ankle ligament complex (LLC) is relatively weak and more susceptible to injury [1]. LLC sprains rank as the most frequently reported diagnosis among athletes in the United States. Comprising three groups of fibers, namely, the anterior talofibular ligament (ATFL), posterior talofibular ligament (PTFL), and calcaneofibular ligament (CFL), the LLC plays a pivotal role. Notably, ATFL is responsible for 66-80% of ankle sprains, while combined ATFL and CFL ruptures account for 20-30% of lateral ankle injuries. In contrast, PTFL injuries occur with lesser frequency. Conflicting evidence indicates that isolated ATFL or combined ATFL-CFL ruptures relate to subtalar instability, impacting ankle stability [2]. Orthopedic practitioners employ various surgical techniques, such as direct repair or autograft/allograft reconstruction of ATFL, with the aim of restoring normal ankle joint stability. Hence, a comprehensive understanding of the anatomical localization, interrelationships, and potential variations within lateral ankle ligaments, especially ATFL, CFL, and PTFL, is of paramount importance [3]. Additional ligaments, namely, the anterior inferior talofibular ligament (AiTFL) and posterior inferior talofibular ligament (PiTFL), are situated at the distal tibiofibular joint. The Brostrom technique stands as the gold standard approach, yielding satisfactory outcomes. Despite adopting a multimodal treatment strategy for this condition, inadequate results have been documented in 21-24% of cases, with 16% exhibiting instability. These less favorable outcomes could potentially be attributed to anatomical variabilities [4]. To achieve a comprehensive understanding of the anatomy and morphometry of lateral ankle ligaments, encompassing ATFL, CFL, PTFL, AiTFL, and PiTFL, this study was undertaken within the Indian population.

## Materials and methods

The ethical committee's approval was secured (Ref. No.: T/IM-NF/Anatomy/23/63), and 15 embalmed adult human cadavers of Indian origin were chosen. These cadavers had no history of limb injuries, and no scar marks were present around the ankle region. The ankle dissection was performed as per Cunningham's dissection manual. The dissection process focused on carefully exposing the anterior and lateral aspects of the ankle, ensuring that over-dissection was avoided to maintain the original ligament and adjacent structure morphology. Precise measurements of ligament length and width were performed using a digital vernier caliper (Yamayo Classic, Tokyo, Japan) with 0.1 mm accuracy.

Each ligament's length was measured at two distinct points: from the center-to-center attachment of ligament fibers (length (a)) and from the farthest attached ligament fibers (length (b)). Ligament width was measured at its midpoint. This measurement protocol was applied to various lateral ankle ligaments, including ATFL, CFL, PTFL, AiTFL, and PiTFL. Additionally, detailed documentation of ligament gross morphology, shape, and orientation was conducted. The classification of ligament complexes was based on the direction of ligament fibers, resulting in categories such as V-shaped, Y-shaped, parallel-shaped, single-band, and double-band. The angles formed between these fibers were measured and recorded. Angulus software was employed to measure specific angles, including the angle between the long axis of the anterior fibular border and the parallel line of the ATFL fiber (angle (a)), the angle between tibiotalar joint lines and the ATFL fiber (angle (b)), and the angle between the long axis of the fibula and the upper CFL fiber (CFL angle). Throughout the measurement process, the ankle was maintained in a neutral position. All the measurements were obtained by two anatomists and averages were taken to eliminate the bias.

## Results

Fifteen formalin-preserved disarticulated lower limbs were selected for the study, with nine on the right side and six on the left side. Observations were made at the attachment site of the ATFL. In 11 out of 15 cases (73.33%), the inner fibers of the ATFL were fused with the capsule of the ankle joint, a condition referred to as intracapsular attachment of the ATFL. The predominant shape observed was a single band of the ATFL, found in eight (53.33%) ankles. Additionally, another shape of the ATFL was identified, resembling a double band (superior and inferior) in seven cases (47.67%). This comprised a V-shaped configuration in three cases (42.86%), a Y-shaped configuration in two cases (28.57%), and a parallel shape in two ankles (28.57%). Obscure ossicles were palpable in four cases (26.67%), and connecting fibers between the ATFL and CFL were present in nine cases (60%). Detailed measurements of the length, width, and angle of the ATFL's single and double bands are provided in Tables 1, 2.

**Table 1:** Anterior talofibular ligament - single-band measurements.

Parameters	Minimum	Maximum	Mean ± SD
Length (a) (mm)	12	18.5	14 ± 2.4
Length (b) (mm)	14	20.6	16 ± 2.2
Width (mm)	4.2	10.7	7.6 ± 2
Angle (a) (degree)	75	138	107 ± 22
Angle (b) (degree)	19	46	30 ± 9.5

**Table 2:** Anterior talofibular ligament (ATFL) - superior and inferior band measurements.

Parameters	ATFL superior band (n = 7)			ATFL inferior band (n = 7)		
	Minimum	Maximum	Mean ± SD	Minimum	Maximum	Mean ± SD
Length (a) (mm)	11	19.3	14.6.8 ± 2.5	7	14	10.8 ± 2.3
Length (b) (mm)	13.7	25	18.50 ± 3.6	9.2	20	13.7 ± 3.8
Width (mm)	4.2	7.3	5.38 ± 1	4	6.4	5.05 ± 0.7

Three distinct morphological shapes of the CFL were observed: a single band in 11 cases (73.33%), a Y-shape in three cases (20%), and a V-shape found in only one case (6.67%). The CFL ligament formed an arch and attached to the ATFL and PTFL in 10 cases (66.67%). Detailed measurements of the length, width, and angle of the CFL are presented in Table 3.

**Table 3:** Calcaneofibular ligament measurements.

Parameters	Minimum	Maximum	Mean ± SD
Length (a) (mm)	10.4	25	18.4 ± 3.9
Length (b) (mm)	12.4	28.7	21.4 ± 4.4
Width (mm)	3.2	8	5.2 ± 1.3
Angle (degree)	109	150	131.1 ± 14.9

The PTFL fibers are interconnected with ligaments such as the ATFL and CFL. In our study, we found that the inferior fascia of the ATFL is connected to the PTFL in 10 cases (66.67%). Furthermore, the PTFL ligament was linked to the CFL ligament in nine (60%) ankles. Detailed measurements of the length and width of the PTFL are provided in Table 4.

**Table 4:** Posterior talofibular ligament - measurements.

Parameters	Minimum	Maximum	Mean ± SD
Length (a) (mm)	14.4	22.7	18.4 ± 2.8
Length (b) (mm)	15.5	28.6	20.9 ± 3.3
Width (mm)	4.3	9.1	6.2 ± 1.4

The AiTFL fibers were associated with the ATFL ligament in two (13.33%) ankles and the PiTFL fibers were connected to the PTFL ligament in three (20%) ankles. Detailed measurements of length and width are presented in Table 5.

**Table 5:** Anterior inferior talofibular ligament and posterior inferior talofibular ligament measurements.

Parameters	Anterior inferior tibiofibular ligament			Posterior inferior tibiofibular ligament		
	Minimum	Maximum	Mean ± SD	Minimum	Maximum	Mean ± SD
Length 1 (mm)	8.8	19	11.7 ± 2.6	9	16.4	12.8 ± 2.1
Length 2 (mm)	11.5	19.1	15.3 ± 2.4	11.5	21.1	16.8 ± 2.8
Width (mm)	7.2	12.1	9.5 ± 1.6	6.2	14.1	10.4 ± 2

Measurements of the lateral ligaments of both the right and left ankles were also conducted. Parameters on the left side of the ankle were found to be greater than those on the right side.

## Discussion

Enhancing understanding of ankle ligament anatomy and morphometry can improve diagnosing ankle sprains and treatment strategies. The present study details the anatomical arrangement, ligament relationships, and fiber orientation of lateral ankle ligaments. Injuries to the LLC stand as some of the most prevalent injuries affecting the lower limb, both in daily life and among athletes [5]. The ankle joint allows for various movements of the foot and ankle, including plantar and dorsiflexion, while inversion and eversion movements transpire at the subtalar joint. These joints are stabilized through a network of diverse ligaments. On the medial aspect, deltoid ligaments are responsible for ankle stability, whereas on the lateral side, the ligaments collectively form the LLC. The ankle complex encompasses the distal tibiofibular syndesmosis, the subtalar joint, and the talocrural joint, all working in concert. These three joints collectively facilitate rearfoot movement, which is often characterized by the following primary planes of motion: frontal plane (inversion-eversion), sagittal plane (plantar flexion-dorsiflexion), and transverse plane (internal rotation-external rotation) [6].

**Figure 1:**
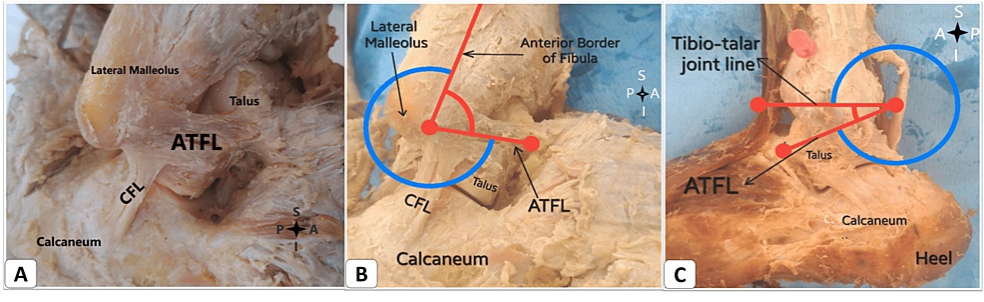
(A) The anterior talofibular ligament (ATFL) and calcaneofibular ligament (CFTL) in the right ankle. (B) Right ankle and (C) left ankle showing the angle (a) and angle (b) measurement in the ATFL. S: superior; I: inferior; A: anterior; P: posterior.

Lateral ankle sprains commonly result from the involvement of one or more ligaments around the ankle joint. These sprains affect both men and women at approximately the same rates. However, a report suggests that female athletes face a 25% higher risk than their male counterparts, likely due to factors such as poor isokinetic strength, ligament laxity, and reduced muscle reaction time [7]. An accurate understanding of lateral ankle ligaments is crucial [8]. In our study, we conducted a morphometric analysis of lateral ankle ligaments, encompassing their shape and size. The ATFL ligament is situated on the lateral side of the ankle, attaching between the lateral malleolus and the talus. The shape and orientation of the ATFL ligament exhibit variability. Based on fiber arrangement, ATFL classifications include V-shaped, Y-shaped, parallel-shaped, single-band, and double-band (superior and inferior) formations. Among these, the most prevalent shape was a single band in eight (53.3%) ankles. The mean length (a) measured 14 mm, while length (b) measured 16 mm. Additionally, the mean width of the ATFL in our study was 7.6 mm. Angle measurements were taken in an ankle-neutral position. Angle (a) was found to be 107 ± 22 degrees, and angle (b) was 30 ± 9.5 degrees, indicating the relationship between tibiotalar joint lines and the parallel lines of ATFL fibers (Figure 1).

The angle between the ATFL and CFL was previously measured falling within the range of 100 to 105 degrees [9]. Notably, we observed inner ATFL fibers merging with the ankle joint capsule, forming intracapsular attachments, present in 11 (73.33%) ankles. In clinical scenarios, these considerations are relevant for ligament repair and anatomical reconstruction. Given the possibility of a single or double bundle of ATFL, reconstruction should extend beyond the single bundle to ensure optimal functional outcomes. Moreover, variations in bundle direction (Y, V, or parallel shapes) should be tailored to individual cases. With discrepancies in bundle numbers and orientations among individuals, an MRI of both normal and injured ankles can guide surgeons toward patient-specific anatomical reconstruction.

During surgery, it is crucial to note the potential involvement of the ankle joint capsule in ligament injuries. Manual palpation revealed obscure ossicles on the fibula in four ankles, serving as reference points for ATFL attachment. These bony landmarks can aid in identifying the origins and insertions of the ATFL and CFL for minimally invasive ankle stabilization procedures. Surgeons must exercise caution during ligament reconstruction surgeries to avoid damaging nearby vessels. A previous study reported an instance of an unusually large perforating branch of the peroneal artery in an 89-year-old female cadaver's left ankle, highlighting the importance of understanding nearby vascular structures [10]. The perforating branch of the peroneal artery might enlarge abnormally and cross in front of the tibiofibular syndesmosis, necessitating preventive measures to avoid vascular injury [10].

In our study, an anastomosis originating from the peroneal artery near the ATFL ligament was identified in four ankles, potentially supplying nearby joint structures. Similar comparable findings were noted in 42 out of 60 ankles (70%) among cases with ATFL double bands [10]. In these instances, the superior and inferior bands were separated by vascular branches originating from the perforating peroneal artery, along with an anastomosis involving the lateral malleolar artery [11]. This observation aligns with the previous literature [12].

**Figure 2:**
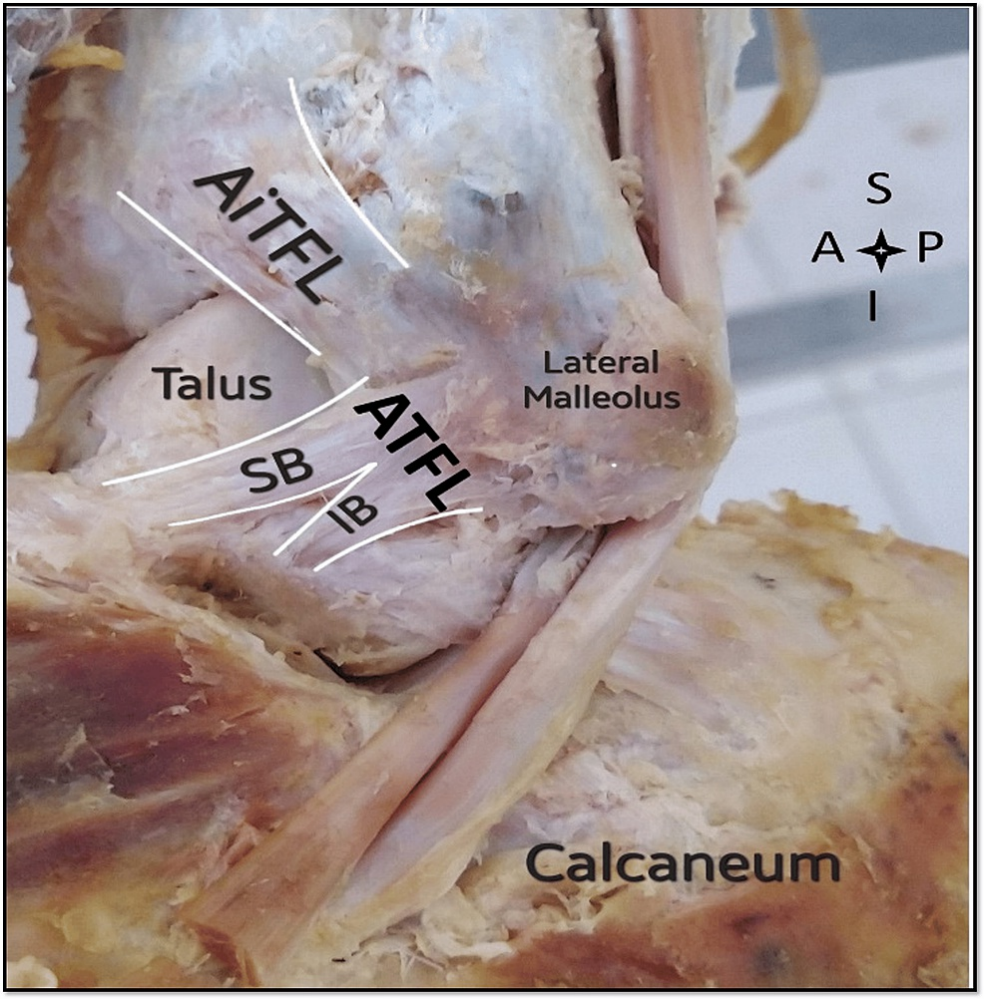
Left ankle with the double band anterior talofibular ligament (ATFL). SB: superior band; IB: inferior band; S: superior; I: inferior; A: anterior; P: posterior; AiTFL: anterior inferior talofibular ligament.

Furthermore, a connection between the ATFL and CFL was observed (Figure 1), also noted in seven (46%) ankles. A similar study was conducted on the LLC, identifying connecting fibers between the ATFL's inferior band and the CFL in 32 ankles [13]. Regarding the ATFL double band (superior and inferior), such a configuration was discovered in seven ankles (Figure 2). The shape was V-shaped in three ankles, parallel in two ankles, and Y-shaped in two ankles.

The CFL ligament connects between the calcaneum and the inferior tip of the lateral malleolus of the fibula. We observed three distinct morphological shapes of the CFL: single band, Y-shape, and V-shape. The most prevalent CFL morphology was the single band, found in 11 (73.33%) ankles, with Y-shaped observed in three ankles and V-shaped in just one ankle. A recent study documented a single bundle in 21 (44.7%) ankles, and a double bundle with Y-shape in 13 (27.7%) ankles [14]. Similarly, morphometric analysis of the CFL was conducted and identified different anatomical shapes, where the single bundle and Y-shape double bundle were the most common, occurring in 21 (44.7%) and 13 (27.7%) ankles, respectively. Less common were the V-shape double bundle and double bundle CFL variants, present in only eight (17%) and five (10.6%) ankles, respectively. The double-bundled, V-shaped, and Y-shaped CFL variants were considered uncommon [15]. The lateral fibula-talocalcaneal ligament complex functions as an anatomical unit, comprising the CFL and the inferior ATFL fascicle. Although separate physical entities, they are linked by arciform fibers and share a common fibular and talar insertion [13]. A study conducted on ATFL bands found the single-banded ATFL in seven out of 14 specimens, while the remaining seven exhibited a double-banded ATFL [16]. The arc-shaped arciform ligamentous fibers originate from the inferior border of the inferior ATFL fascicle and the lateral part of the talar body. These fibers travel distally and posteriorly to the anterior border of the CFL, forming a parabola and joining the inferior ATFL fascicle and the CFL. The observed Y-shaped CFL variant, along with the connecting arc-shaped arciform fibers, may have a relationship with the inferior ATFL fascicle. The arching fibers of the ATFL and CFL, combined with their various shapes, could hold significance in providing stability to the subtalar joint [17]. However, it is worth noting that the observed Y-shape variant is distinct from the others [13,14].

**Figure 3:**
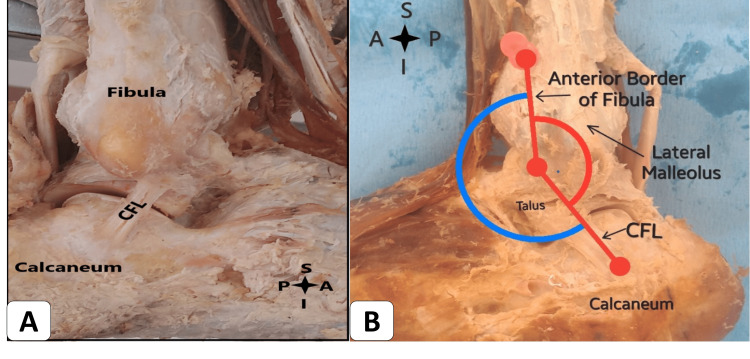
(A) Right ankle with the calcaneofibular ligament (CFL). (B) The CFL angle in the left ankle. S: superior; I: inferior; A: anterior; P: posterior.

**Figure 4:**
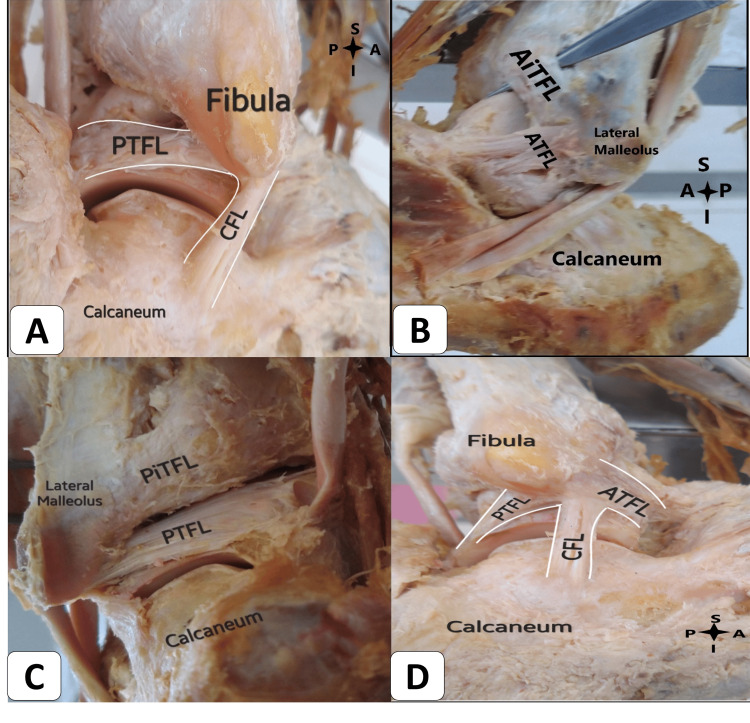
(A) Posterior talofibular ligament (PTFL) and calcaneofibular ligament (CFL) in the right ankle. (B) Anterior inferior tibiofibular ligament (AiTFL) in the left ankle. (C) The posterior view of the left ankle shows the posterior inferior tibiofibular ligament (PiTFL). (D) Anterior talofibular ligament (ATFL), CFL, and PTFL arching fiber sharing the common fibular origin in the right ankle. S: superior; I: inferior; A: anterior; P: posterior.

In our study, we also observed a connection between the CFL and PTFL. The CFL ligament arches and attaches to the ATFL and PTFL in 10 (66.67%) ankles. The angle between the long axes of the anterior border of the fibula and the parallel line of the upper fiber of the CFL measured 131 degrees (Figure 3). The length (a) from the center to the center attachment of the CFL ligament was measured at 18.4 mm. Meanwhile, the length (b), representing the farthest attachment of CFL fibers from the lateral malleolus of the fibula to the talus, measured 21.4 mm. The width of the CFL, measured at its midpoint, was found to be 5.2 mm. Another study reported a length of 29.9 ± 1.4 mm, which is notably longer [14]. Notably, a systematic review indicated substantial variation in CFL length, ranging from 18.5 to 35.8 mm, potentially stemming from differing reference insertion points across studies [18]. PTFL is responsible for attaching the lateral malleolus to the talus posteriorly (Figure 4). In our study, the PTFL ligament exhibited a connection with the CFL in nine (60%) ankles. The measured length of the PTFL was 20.9 mm, while the width was 6.2 mm. In a study by Burks and Morgan, a longer length of 24.1 mm was reported, along with a width of 6.9 mm [19].

The AiTFL connects the lower tibia and fibula frontward. Its shape is triangular. Distal tibiofibular syndesmosis injury occurs in 1-11% of ankle sprains. After six months, 40% of patients still complain of ankle instability [20]. This can result from broadened ankle mortise due to stretched syndesmotic ligaments following an acute ankle sprain. Harris and Fallat (2004) and Ramsey and Hamilton (1976) noted that increasing ankle mortise width by 1 mm reduces tibiotalar joint contact area by 42%. This reduction may induce instability and early tibiotalar joint osteoarthritis [21].

In our study, AiTFL fibers linked with the ATFL ligament in two (13.33%) ankles. The connected AiTFL fiber's average length was 15.3 ± 2.4 mm (11-19.1 mm), and the width was 9.5 ± 1.6 mm (7.2-12.1 mm). Ebraheim (2006) measured length at 10.4 ± 3.1 mm (8.2-12.6 mm), and the width was 17.7 ± 1.0 mm (17.1-18.5 mm) at the tibial side, perhaps due to geographical variations. Nikolopoulos et al. (2004) had slightly different measurements (length: 3-6 mm; thickness: 2-4 mm; width: 2-4 mm). The PiTFL fibers were connected with the PTFL ligament in three (20%) ankles. PiTFL fibers connected with the PTFL ligament in three (20%) ankles. PiTFL has a similar shape and structure as AiTFL, with its inner fascia converging with the fibula posteriorly. The mean PiTFL length was 16.8 ± 2.8 mm (11.5-21.1 mm). Nikolopoulos et al. (2004) and Ebraheim et al. (2006) reported 21.8 ± 7.5 mm and 6.4-32.5 mm length. PiTFL is multifascicular with multiple collagen bundles. Some fibers touch the transverse ligament [22]. It acts as a "spring," allowing slight separation between medial and lateral malleoli during dorsiflexion at the talocrural joint, aiding talus wedging in the mortise [23]. Ultrasonography reflects this action during foot flexion, showing tension in AiTFL and PiTFL [24].

Right and left ankle ligament measurements were conducted. The ATFL ligament's length (a) on the right side was 14.4 mm, while on the left side, it was 14.3 mm. Length (b) measured 17.2 mm on the right side and 18.2 mm on the left side. The width was 6.5 mm on both sides. The angle (a) on the right side was 104 degrees, whereas on the left side, it measured 108 degrees. Similarly, angle (b) measured 34 degrees on the right side and 27 degrees on the left side. The ATFL's inferior band length was 12.3 mm on the right side and 15.5 mm on the left side. The farthest attachment length of AiTFL was 14 mm on the right side and 16 mm on the left side. The attachment fibers of PiTFL measured 11 mm on the right side and 14 mm on the left side. All measurements on the left side exceeded those on the right side. The CFL length (a) measured 20.8 mm on the right side and 22.4 mm on the left side. The angle between the anterior border of the fibula's long axes and the parallel line of the CFL measured 133 degrees on the right side and 127 degrees on the left side. Except for the angle, all measurements on the left side were larger than those on the right side.

Limitations of study

The study was conducted on formalin-embalmed cadavers, where there is a possibility of contracture or stiffness, contributing to minimal variations in measurements compared to a living person. Additionally, it is noteworthy that the study involved a limited number of specimens. Conducting a multicentric study covering a larger sample size would be instrumental in obtaining more robust and generalizable data, enhancing the validity and applicability of the findings to a broader population.

## Conclusions

The study findings hold promise for surgeons, providing an anatomical guide to lateral ankle ligaments through identifiable osseous landmarks for reconstruction. Distinct morphological variations, including double bundle, V-shaped, and Y-shaped variants, were revealed. Their interaction with the lateral talocalcaneal ligament and inferior ATFL fascicle offers insights for surgery. Moreover, this study highlights instances where ATFL, CFL, and PTFL converge at the inferior lateral malleolus via continuous connective fibers. Understanding their anatomy, relative positions, and relations aids palpation and ultrasonography. Details of AiTFL, PiTFL, and tibiofibular syndesmosis benefit surgeons and radiologists. Furthermore, these data enhance graft sizing, selection, and anatomical placement in surgical reconstruction. This study not only advances surgical techniques but also deepens comprehension of lateral ankle ligaments, benefiting clinical practice and research.
